# Supplementary material for: Deep sequencing of circulating exosomal microRNA allows non-invasive glioblastoma diagnosis
Source: NPJ Precis Oncol. 2018 Dec 12;2:28. doi: 10.1038/s41698-018-0071-0 (PMC6290767; doi:10.1038/s41698-018-0071-0)

Supplementary Fig. 1: Ingenuity Pathway Analysis revealed significant overlap with Glioblastoma signaling pathway (*p*-value=3.36E^{-12})

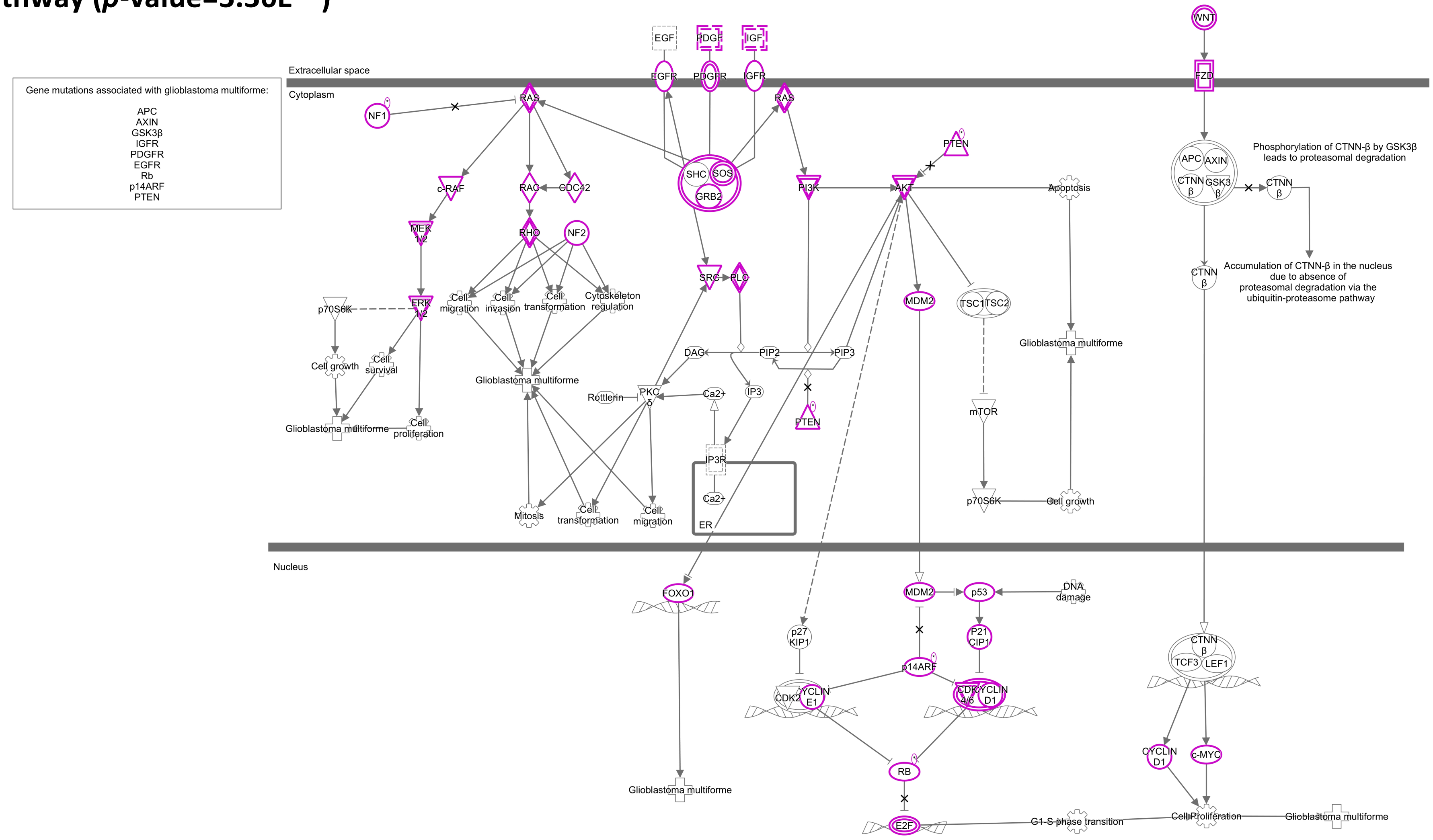

Glioblastoma signaling pathway annotated with molecules targeted by significant, differentially expressed exosomal miRNAs (in magenta).

Supplementary Fig. 2A: Box-and-whisker plots, differentially expressed miRNAs in GBM *vs*. healthy controls

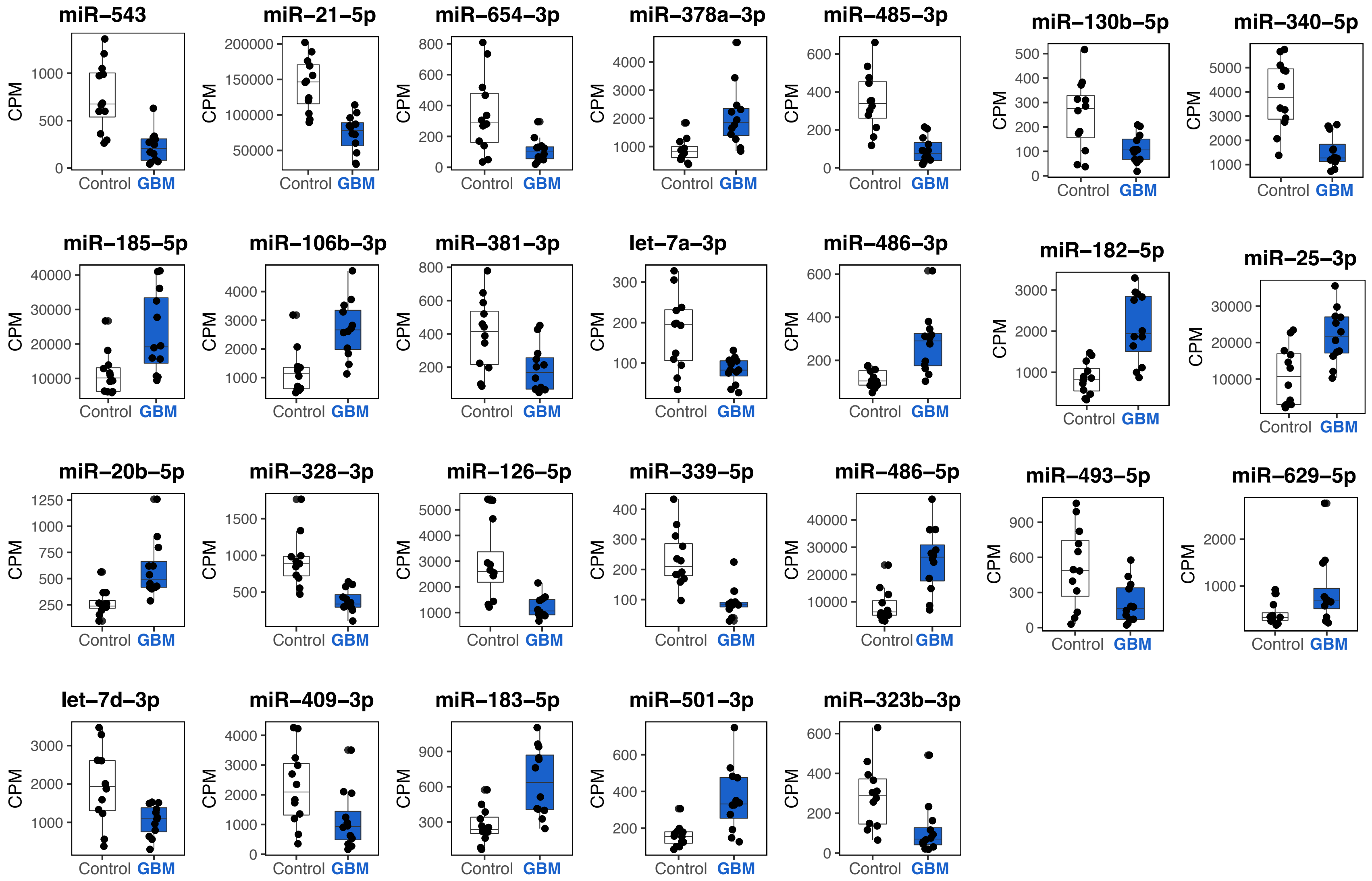

Supplementary Fig. 2B: ROC curves, differentially expressed miRNAs in GBM *vs.* healthy controls

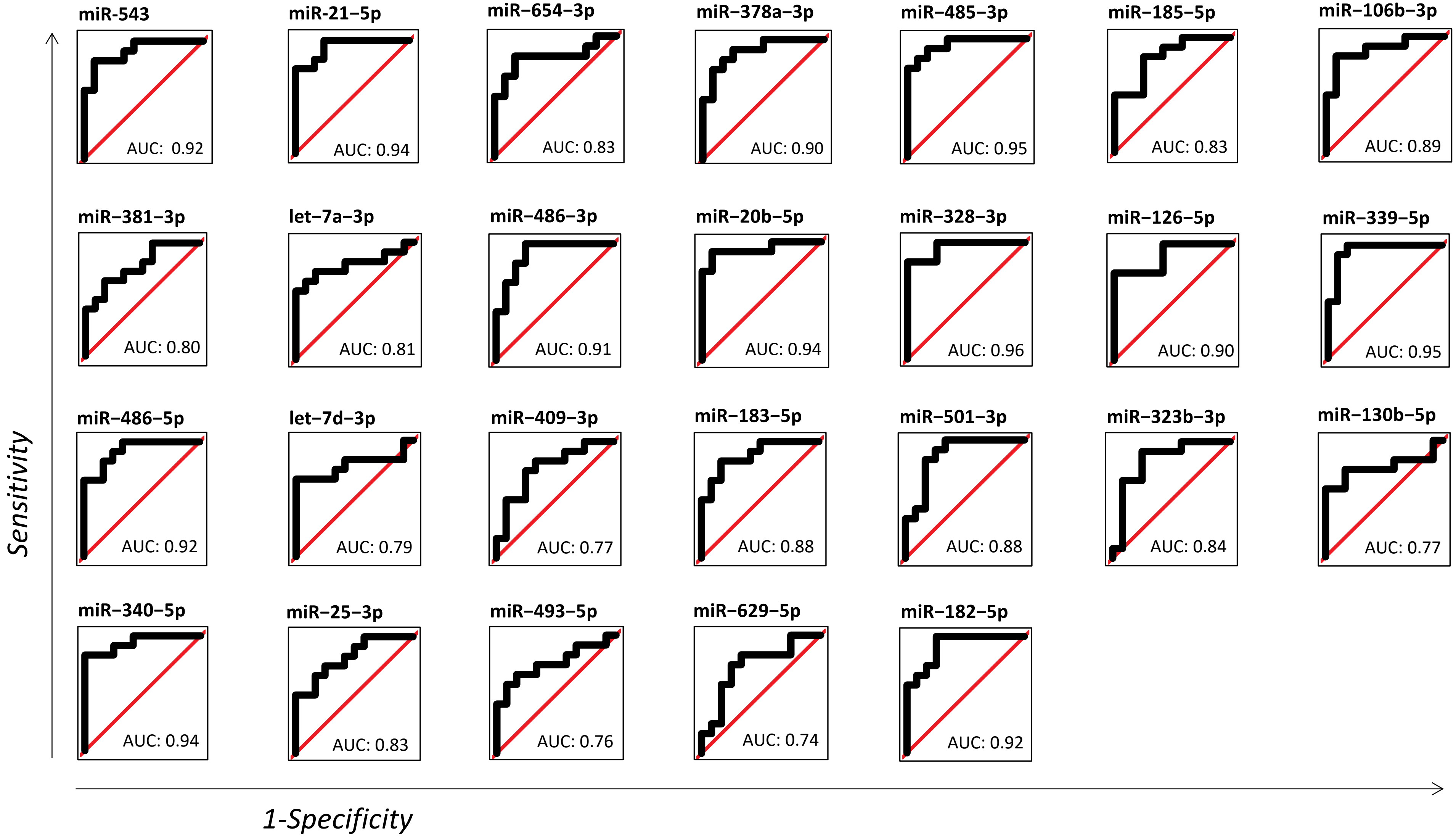

Supplementary Fig. 3A: Box-and-whisker plots, differentially expressed miRNAs in GII-III *vs*. healthy controls

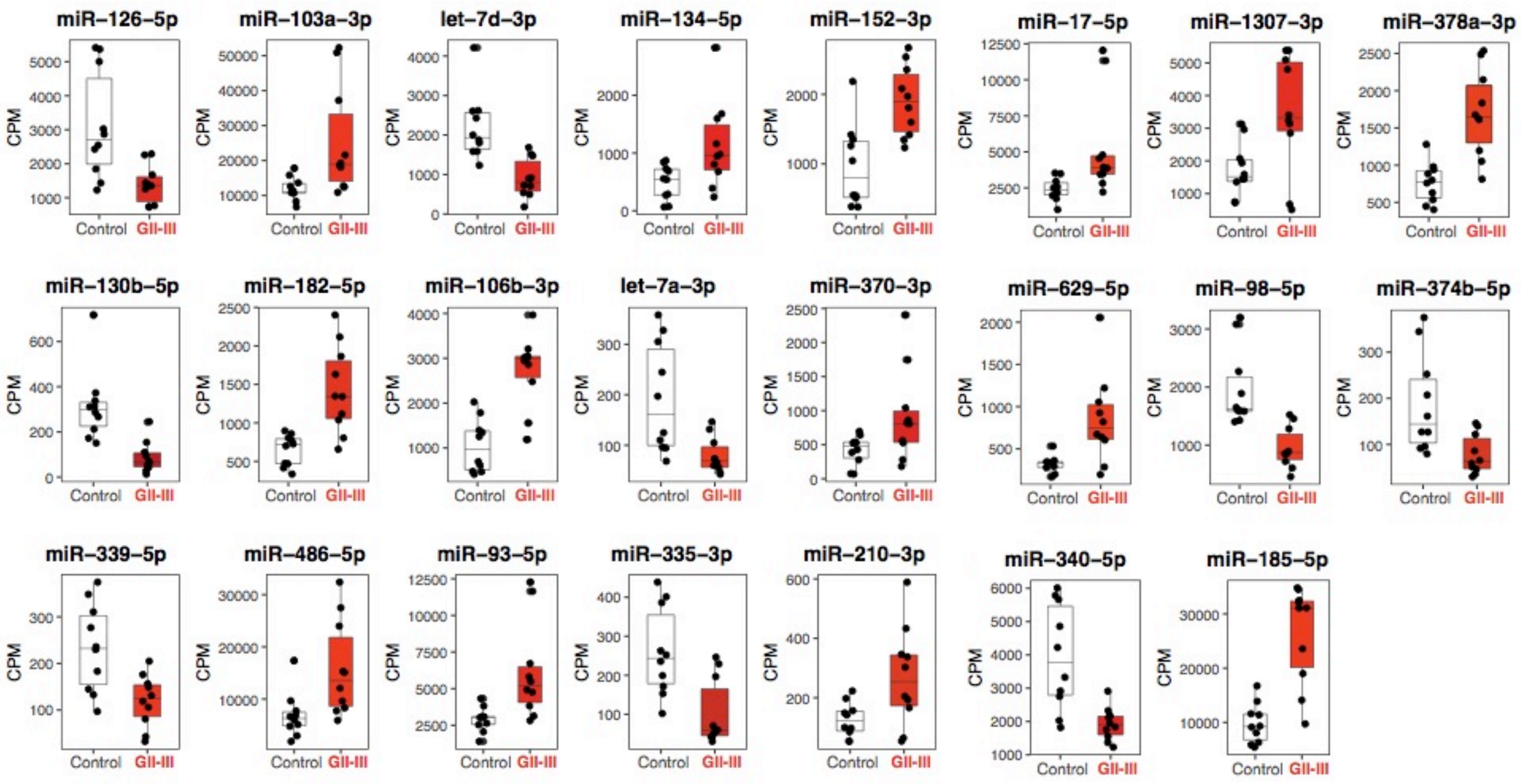

Supplementary Fig. 3B: ROC curves, differentially expressed miRNAs in GII-III *vs*. healthy controls

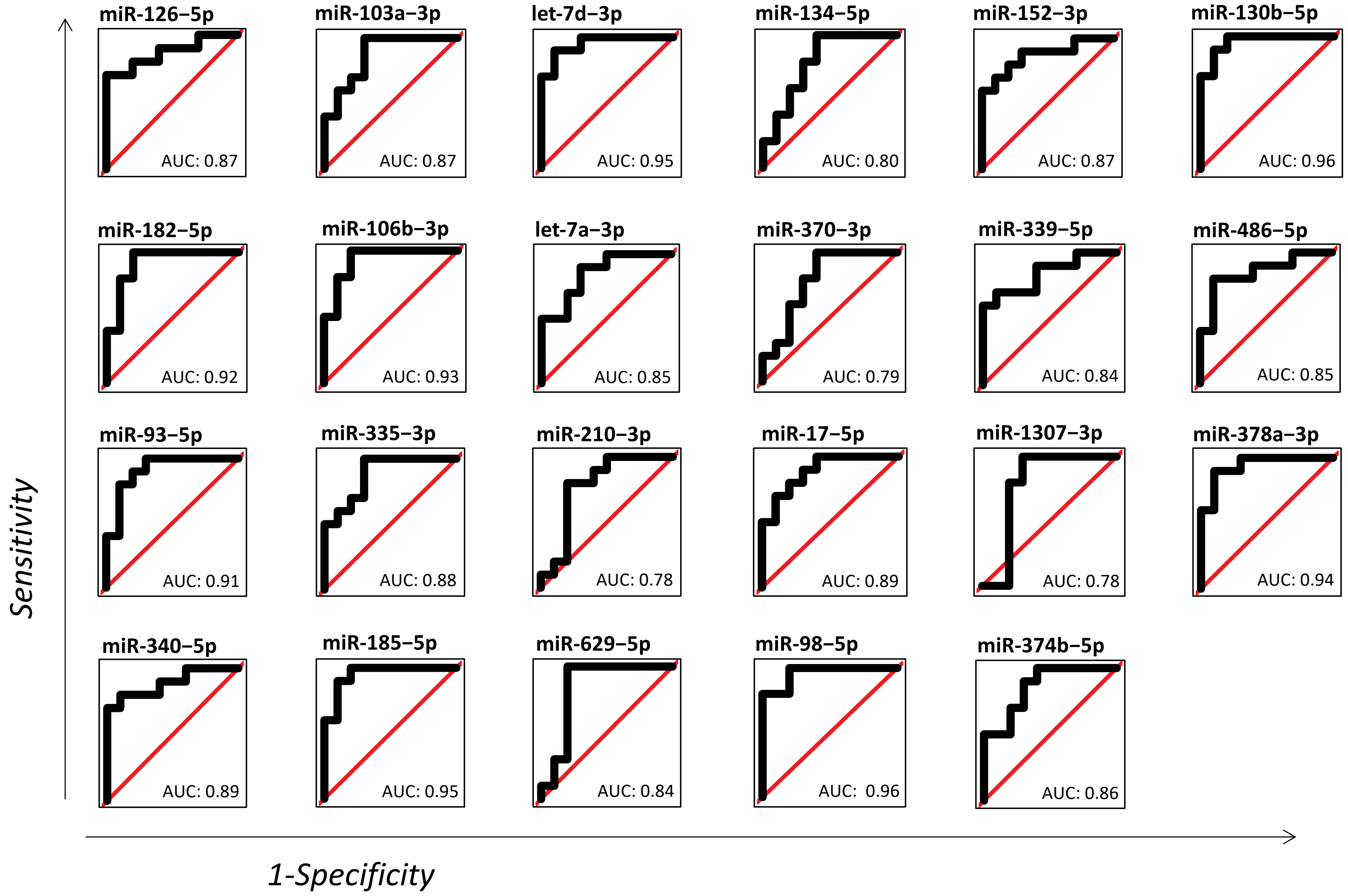

Supplementary Fig. 3C: Partitioning and Random Forest Modeling to select stable miRNAs for GII-III *vs*. healthy controls

Random Forest after partitioning, Importance of Contribution

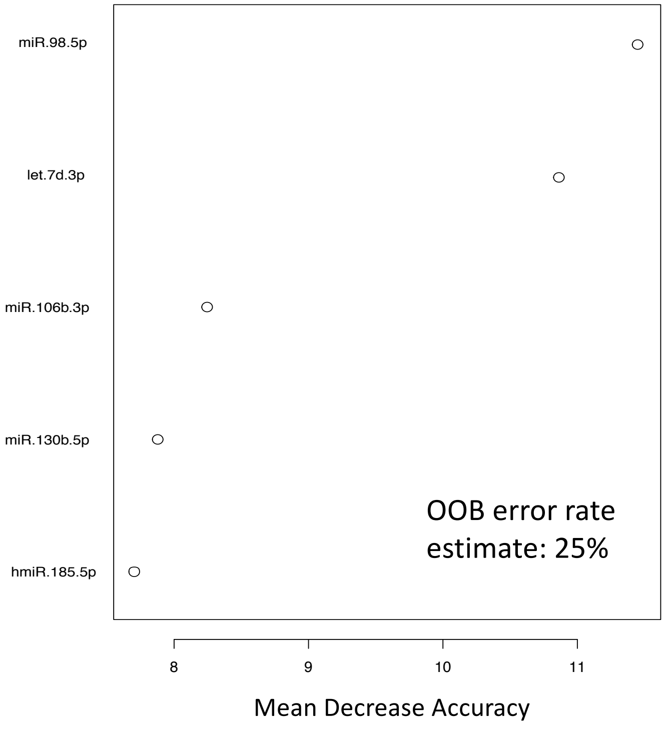

Violin plot of error distributions of stratified miRNA signature size

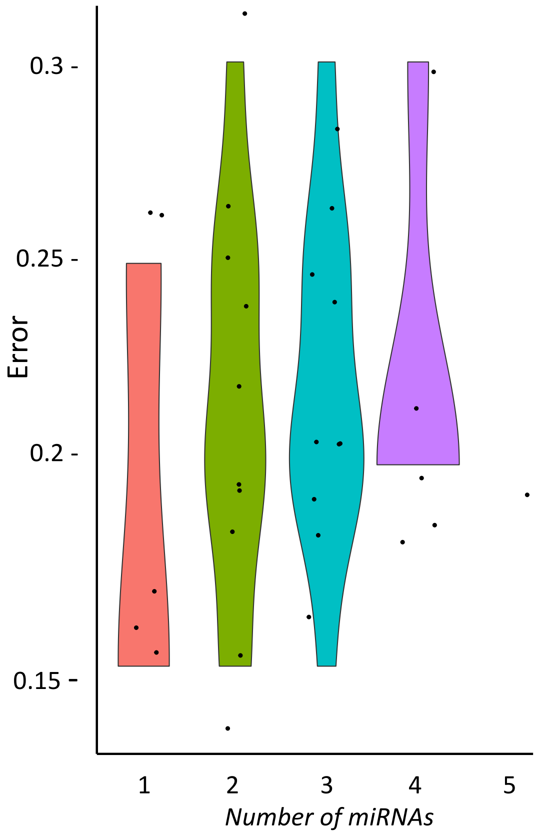

Combinations of miRNAs with lowest OOB error rate

**# miRNA**	**miRNA combination**	**Error**
2	miR-106b-3p, miR-98-5p	0.132
2	miR-106b-3p, miR-130b-5p	0.158

Supplementary Fig. 4A: Box-and-whisker plots, differentially expressed miRNAs in GII-III *vs*. GBM

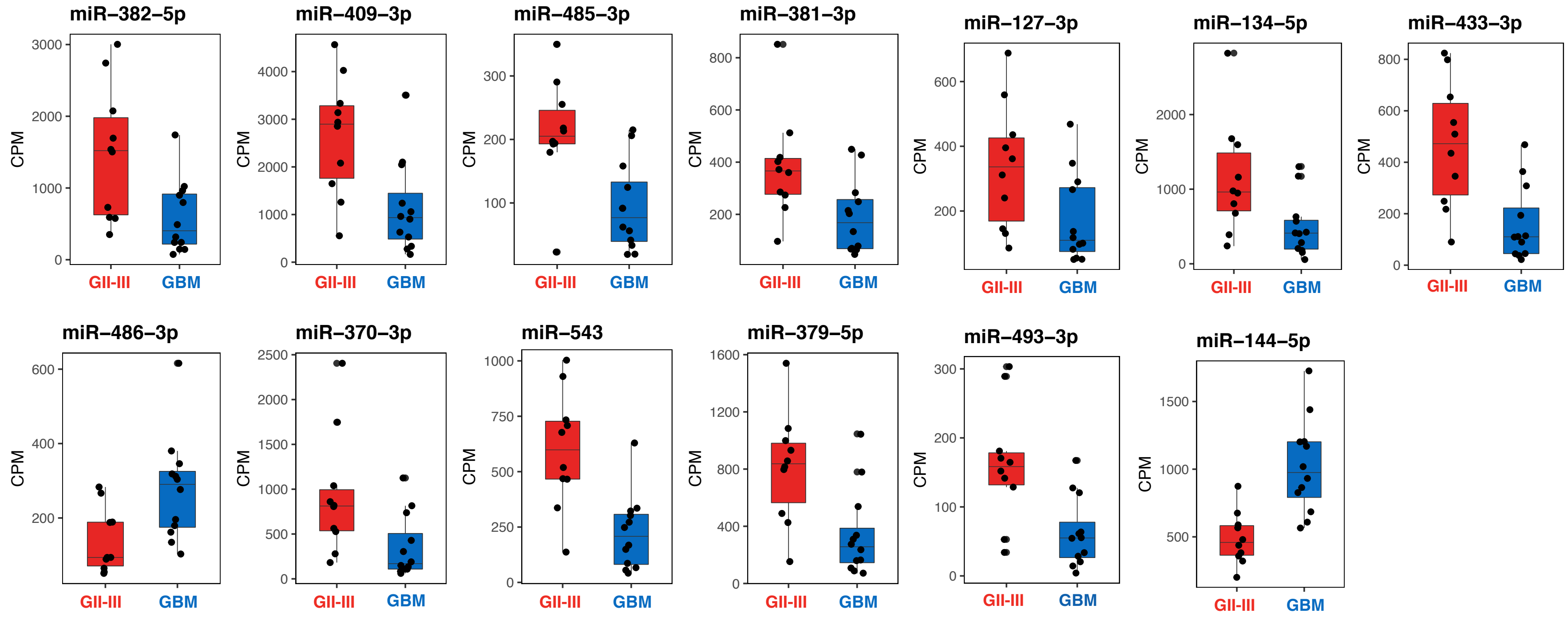

Supplementary Fig. 4B: ROC curves, differentially expressed miRNAs in GII-III *vs.* GBM

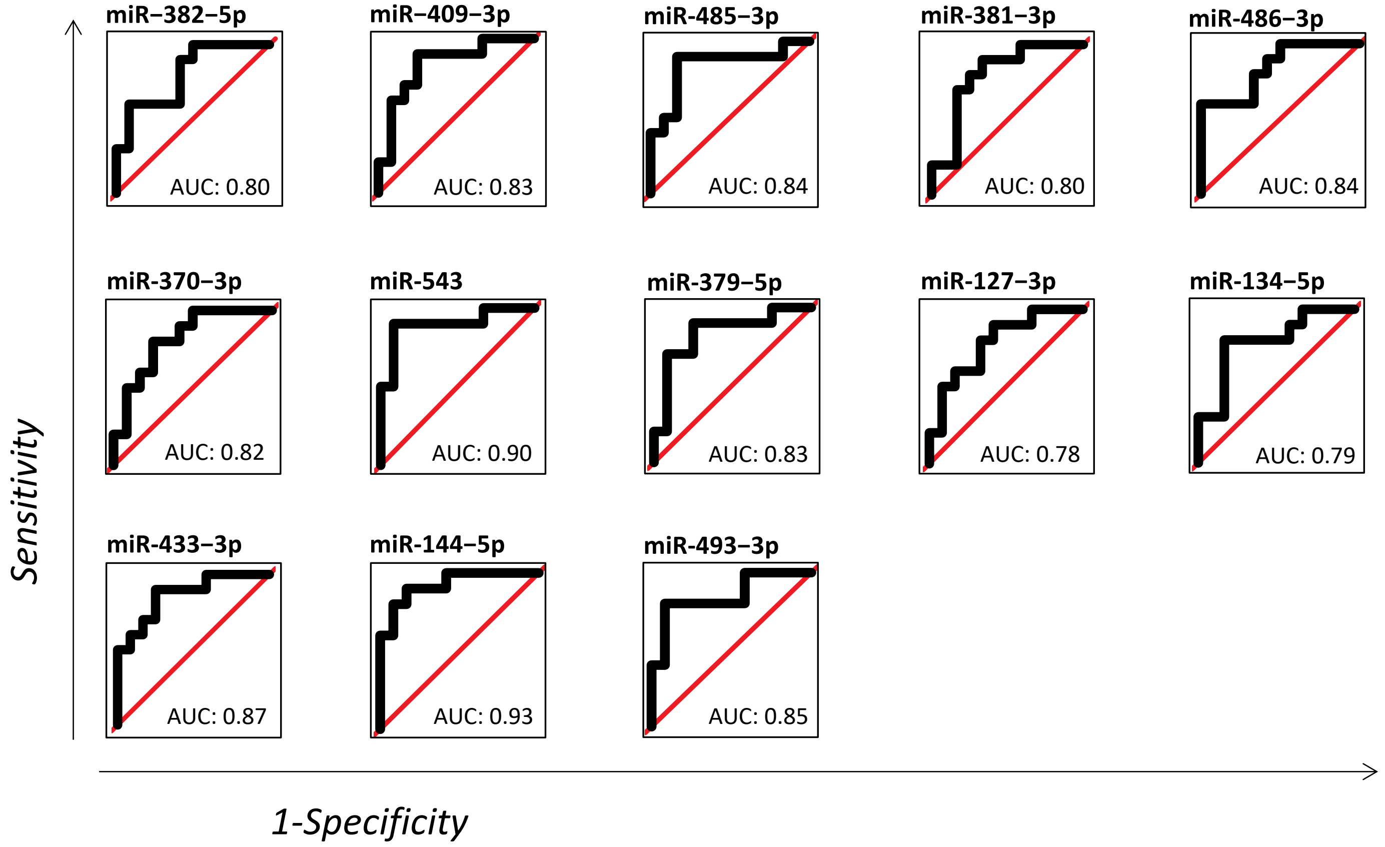

Supplement: Supplementary file 1 — Supplementary Figures [file 41698_2018_71_MOESM1_ESM.pdf]
